# Lung-protective ventilation suppresses systemic and hepatic vein levels of cell-free DNA in porcine experimental post-operative sepsis

**DOI:** 10.1186/s12890-020-01239-y

**Published:** 2020-07-31

**Authors:** Axel Nyberg, Alexander Larsson, Juulia Jylhävä, Mikko Hurme, Jesper Sperber, Miklós Lipcsey, Markus Castegren

**Affiliations:** 1 Department of Anaesthesiology & Intensive Care, Centre for Clinical Research, Sörmland, Uppsala University, Mälarsjukhuset, SE-631 88 Eskilstuna, Uppsala, Sweden; 2 Department of Medical Sciences, Uppsala University, Uppsala, Sweden; 3 Centre for Clinical Research, Region of Västmanland, Uppsala University, Uppsala, Sweden; 4 Department of Medical Epidemiology and Biostatistics, Karolinska Institute, Stockholm, Sweden; 5 Faculty of Medicine and Health Technology, Tampere University, Tampere, Finland; 6 Hedenstierna laboratory, CIRRUS, Anesthesiology and Intensive Care, Department of Surgical Sciences, Uppsala University, Uppsala, Sweden; 7 Perioperative Medicine and Intensive Care (PMI), Karolinska University Hospital and CLINTEC, Karolinska Institute, Stockholm, Sweden

**Keywords:** Innate immunity, Cytokines, Endotoxin, Low tidal volume ventilation, Intensive care

## Abstract

**Background:**

Plasma levels of cell**-**free DNA (cf-DNA) are known to be elevated in sepsis and high levels are associated with a poor prognosis. Mechanical ventilation affects systemic inflammation in which lung-protective ventilation attenuates the inflammatory response. The aim was to study the effect of a lung protective ventilator regime on arterial and organ-specific venous blood as well as on trans-organ differences in cf-DNA levels in a porcine post-operative sepsis model.

**Method:**

One group of anaesthetised, domestic-breed, 9–12 weeks old, pigs were ventilated with protective ventilation (V_T_ 6 mL x kg^− 1^, PEEP 10 cmH_2_O) *n* = 20. Another group, ventilated with a medium high tidal volume and lower PEEP, served as a control group (V_T_ 10 mL x kg^− 1^, PEEP 5 cm H_2_O) *n* = 10. Blood samples were taken from four sources: artery, hepatic vein, portal vein and, jugular bulb. A continuous endotoxin infusion at 0.25 μg x kg^− 1^ x h^− 1^ for 5 h was started following 2 h of laparotomy, which simulated a surgical procedure. Inflammatory cytokines and cf-DNA in plasma were analysed and trans-organ differences calculated.

**Results:**

The protective ventilation group had lower levels of cf-DNA in arterial (*p* = 0.02) and hepatic venous blood (*p* = 0.03) compared with the controls. Transhepatic differences in cf-DNA were lower in the protective group, compared with the controls (*p* = 0.03). No differences between the groups were noted as regards the transcerebral, transsplanchnic or the transpulmonary cf-DNA differences.

**Conclusions:**

Protective ventilation suppresses arterial levels of cf-DNA. The liver seems to be a net contributor to the systemic cf-DNA levels, but this effect is attenuated by protective ventilation.

## Background

Circulating cell-free DNA (cf-DNA) are fragments of DNA found in the blood of healthy and diseased individuals, with increased levels in the diseased state [1, 2]. Over the past decades, increased levels of cf-DNA have been detected in the plasma of patients with diseases e.g., cancer [1], trauma [3], stroke [4], myocardial infarction [5, 6], post-cardiac arrest [7], and pancreatitis [8], with higher levels associated with worse outcome.

High levels of cf-DNA are found in the plasma of patients with sepsis and septic shock, where it may have, a diagnostic and prognostic role. One study reported increased levels of cf-DNA in patients with infection as a cause of fever, as opposed to a non-infectious cause [9]. Several other studies have demonstrated higher levels of cf-DNA in non-survivors compared with survivors in sepsis, and septic shock [2, 10–13]. The presence of elevated concentration levels of cf-DNA in animal sepsis models has also been demonstrated [14, 15]. The exact origin of cf-DNA is not known but current evidence points towards apoptotic and necrotic host cells as the source [10, 16, 17]. It has also been proposed that cf-DNA is released by activated neutrophils [18, 19], to form neutrophil extracellular traps. However, this view has recently been challenged [14].

Mechanical ventilation (MV) can be a lifesaving intervention in the face of impending respiratory failure, to protect the airway or rest fatigued respiratory muscles. Injudicious use of MV with moderate to high tidal volumes has been proven harmful in certain circumstances [20, 21]. Experimental studies also suggests it is involved in extra-pulmonary organ failure [22]. Imai et al., using a rabbit model of hydrochloric acid aspiration, showed that high tidal volumes induced apoptosis in distant organs, most notably in the small intestine and kidney [23]. In sepsis, the pro-inflammatory response may be involved in the development of multiorgan dysfunction syndrome (MODS) [24]. After the initial hyper-inflammatory state, sepsis is characterised by immunosuppressive responses in which apoptosis and necrosis of inflammatory cells seem to be a contributing factor [25, 26]. Our group has recently shown that the liver is a major contributor to the systemic levels of tumor necrosis factor alpha (TNF-α). In the same study, protective ventilation with low tidal volumes (6 mL x kg^− 1^), in comparison with medium high tidal volume ventilation (10 mL x kg^− 1^), suppressed the overall cytokine levels, particularly TNF-α in the liver [27]. This led to the hypothesis that cf-DNA levels would be affected by protective ventilation in an experimental sepsis model. The porcine model used is a large animal intensive care model, resembling the clinical scenario of human intensive care.

The primary endpoint was to investigate whether protective ventilation with low tidal volumes and higher positive end expiratory pressure (PEEP) (V_T_ 6 mL x kg^− 1^, PEEP 10 cmH_2_0) suppressed systemic and organ-specific plasma levels of cf-DNA compared with medium tidal volume ventilation and lower PEEP (V_T_10 mL x kg^− 1^, PEEP 5 cmH_2_0in a post-operative porcine model of sepsis. Secondary endpoints were to investigate the trans-organ differences in cf-DNA and inflammatory cytokines in the liver, brain, splanchnic organs, and lungs.

## Methods

### Animals

The animals included in this study were also studied in conjunction with another study previously published [27]. Thirty domestic-breed piglets of both sexes with a weight of 28.5 ± 1.5 (mean ± SD) kg were included. All animals, aged 9–12 weeks, were apparently healthy and sexually immature.

### Ethical statement

The study was designed with consideration of Minimum Quality Threshold in Pre-clinical Sepsis Studies (MQTiPSS) [28] and reported in adherence to the Animal Research: Reporting of In Vivo Experiments (ARRIVE) guidelines [29]. The study was approved by the Animal Ethics Board (Uppsala djurförsöksetiska nämnd, permit no. C250/11) in Uppsala, Sweden. The animals were acquired from a private source, Mångsbo Gård, Uppsala, Sweden. Handling of the animals was done according to the guidelines of the Swedish Board of Agriculture. All measures were taken to decrease suffering. The animals were allowed to eat and drink provided ad libitum up to 1 h (h) before the start of the experiment. All surgical procedures were performed under general anaesthesia and signs of pain were monitored and treated. Immediately after the experimental endpoint, the animals were killed by an intravenous (i.v.) injection of potassium chloride and MV withdrawn.

### Anaesthesia

Before arrival at the research facility, all animals were premedicated with an intramuscular injection of 50 mg xylazine. The animals were studied in groups of two per day. General i.v. anaesthesia was induced with zolazepam 3 mg x kg^− 1^, tiletamine 3 mg x kg^− 1^, xylazine 2.2 mg x kg^− 1^ and atropine 0.04 mg x kg^− 1^. A bolus i.v. injection of ketamine 100 mg and morphine 20 mg was administered before securing the airway by surgical tracheostomy. Thereafter, MV (Servo 900C or Servo I, Siemens Elema, Stockholm, Sweden) was introduced and continued until the end of the experiment. Anaesthesia was maintained by a continuous i.v. infusion of sodium pentobarbital 8 mg x kg^− 1^ x h^− 1^, morphine 0.26 mg x kg^− 1^ x h^− 1^ and pancuronium bromide 0.48 mg x kg^− 1^ x h^− 1^, dissolved in a glucose solution of 2.5% concentration, resulting in a fluid administration rate of 15 mL x kg^− 1^ x h^− 1^.

### Surgery

After bilateral paratracheal skin incisions the thyroglossus arteries and jugular veins were identified with blunt dissection. A branch of the right thyroglossus artery was cannulated using a 5F catheter to sample blood and monitor blood pressure. A central venous catheter and a 7F pulmonary artery catheter were then placed via the right external jugular vein. On the left side, the internal jugular vein was cannulated using a 5F arterial catheter advanced 5 cm cranially to approximate the jugular bulb. A 7F Swan-Ganz catheter was advanced, via the left external jugular vein, under fluoroscopic guidance into a hepatic vein. To access the portal vein, a 10 cm skin incision was made under the left costal margin. The muscles, fascia, and peritoneum were bluntly dissected to reach the splenic vein, which was cannulated close to the splenic hilum using a 5F arterial catheter and then further advanced 15 cm. To confirm catheter tip position in the hepatic and portal vein 5 mL iohexol (OmnipaqueTM, GE Healthcare AB, Stockholm, Sweden) solution was injected under fluoroscopy. Lastly, the urinary bladder was catheterised through a small laparotomy.

After the preparatory surgery, an i.v. fluid bolus of Ringer’s acetate 20 mL x kg^− 1^ was administered and a stabilisation time of 30 min allowed, after which baseline physiological values were recorded, blood samples collected, and the abdominal fascia and skin sutured. A lung recruitment manoeuvre was performed by a stepwise increase of PEEP until an inspiratory plateau pressure of 30 cmH_2_O was achieved and then inspiratory pressure was kept constant for 10 s.

### Protocol

**Fig. 1:**
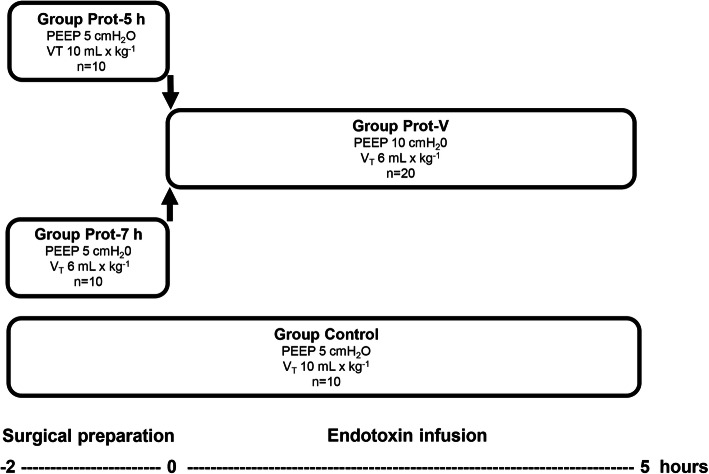
Design of the study

The animals were randomised in blocks of three groups, Fig. 1. Two groups received protective ventilation, Prot-5 hand Prot-7 h (both *n* = 10), and a Control group, (*n* = 10). After baseline, both the protective ventilation groups were ventilated with a low tidal volume (V_T_) of 6 ml x kg^− 1^, a PEEP set to 10 cmH_2_O, and a respiratory rate (RR) of 35 while the control group had a V_T_ of 10 ml x kg^− 1^, a PEEP of 5 cmH_2_O and a RR of 25 in order to achieve the same minute ventilation as the group with protective ventilation. The protective ventilation groups only differed in ventilation during the preparatory phase before baseline, a time period of 2 h. During this period, the group Prot-5 h was ventilated with a V_T_ of 10 mL x kg^− 1^ and a PEEP of 5 cmH_2_0, whereas the group Prot-7 hwas ventilated with a V_T_ of 6 mL x kg^− 1^ and a PEEP of 5 cmH_2_0. Volume control mode with an inspiratory to expiratory (I:E) ratio of 1:2 and a fraction of inspired oxygen (FiO_2_) of 0.3 was used in all groups.

At 0 h an i.v. infusion of endotoxin was started at 0.25 μg x kg^− 1^ x h^− 1^ for all animals, independent of experimental group, and kept for 5 h, which was the predetermined time of the experiment. Slow i.v. injection of cefuroxime 20 mg x kg^− 1^ was administered at 1 h to prevent bacterial contamination of the model.

### Interventions

Because the experiment was set-up to mimic an intensive care setting of post-operative sepsis, a goal-directed intervention protocol was used. Respiratory settings adjusted during the experiment were the FiO_2_ and the RR. FiO_2_ was adjusted by increments of 0.1 or decrements of 0.05 to maintain partial pressure of arterial oxygen (PaO_2_) within 12 to 18 kPa. RR was increased or decreased by 10% to keep arterial levels of carbon dioxide (PaCO_2_) between 5 and 5.5 kPa.

For circulatory management during the experiment, it has to be considered that pulmonary hypertension frequently occurs in porcine models after initiation of endotoxin infusion, most often quickly reversible [30, 31]. Therefore, if mean arterial pressure (MAP) approximated mean pulmonary arterial pressure (MPAP) within the first 90 min an intravenous dose of adrenalin 0.1 mg was administered. This dose was repeated once if necessary. If MAP equalled MPAP after more than 90 min since the start of the endotoxin infusion, a 1 mL i.v. bolus of noradrenalin 20 μg x mL^− 1^ was administered and followed by a continuous i.v. infusion of noradrenaline 20 μg x mL^− 1^ at a rate of 5 mL x h^− 1^. The bolus was repeated, and the infusion rate doubled if MAP was equal or near equal to MPAP. In addition to the basic fluid protocol, isolated MAP values of < 50 mmHg were treated with an i.v. fluid bolus of Ringer’s acetate 10 ml x kg^− 1^ up to a maximum rate of 15 ml x kg^− 1^ x h^− 1^. The additional fluid was only administered 90 min after the start of the endotoxin infusion.

### Analyses

Blood, sampled in sodium heparin from the artery, jugular bulb, portal, and hepatic vein, was taken at 0, 1, 3, and 5 h to analyse cf-DNA, TNF-a, interleukin 6 (IL-6), and interleukin 10 (IL-10). The samples were centrifuged to retain plasma and immediately frozen for later analyses. Total cf-DNA was measured directly in plasma using a Quant-iT™ DNA High-Sensitivity Assay kit and a Qubit® fluorometer (Invitrogen, Carlsbad, CA, USA) according to the manufacturer’s instructions. The Quant-iT™ DNA High-Sensitivity Assay is based on a DNA-intercalating dye, which measures all double-stranded cf-DNA, regardless of its sequence, fragment size, or integrity. This method requires no prior DNA extraction step, hence, the difficulties related to capturing and recovering cf-DNA fragments of all sizes with one DNA extraction kit can be avoided. Each sample was analysed in duplicate, and the mean of the two measurements was used as the final value. The lower level of detection was 0.2 μg x mL^− 1^. At the mean cf-DNA level of 0.481 μg x mL^− 1^, the intra- and inter-day variation coefficients for the Quant-iT™ DNA High-Sensitivity Assay were 2.64 and 5.37%, respectively. Commercial porcine-specific sandwich enzyme-linked immunosorbent assay (ELISA) was used to determine TNF-α, IL-6, and IL-10 in plasma (DY690B (TNF-α) and DY686 (IL-6), R&D Systems, Minneapolis, MN, USA and KSC0102 (IL-10), Invitrogen, Camarillo, CA, USA). The lower detection limits in sodium heparin plasma were 230 pg × mL^− 1^ for TNF-α, 60 pg × mL^− 1^ for IL-6 and 60 pg × mL^− 1^ for IL-10. All ELISAs had intra-assay coefficients of variation (CV) of < 5% and total a CV of < 10%.

### Endpoints, calculations, and statistics

Based on previous studies, the power analysis was calculated on a detectable difference of 15% of cytokines in systemic plasma at the experimental endpoint, an alpha error of 0.05, a power of 0.8, and an SD of 10%, yielding at least six evaluable animals per group. The groups were expanded to 10 animals in the current experiment to capture potential differences in organ-specific locations where we had no previous experience of cf-DNA levels. To study the effects of tidal volume and PEEP of plasma levels on cf-DNA with, and without, systemic inflammation, the three-group study design was deemed warranted, Fig. 1. A priori to the main data analyses, we decided to investigate whether there was an effect of different tidal volume and PEEP *before* baseline on cf-DNA levels when comparing groups Prot-5 h with Prot-7 h. If no trend towards such an effect was noted, the two groups were to be combined in the main analyses to increase power and reduce the number of animals. The cutoff value for what was considered a trend towards a difference between groups Prot-5 hwithProt-7 h, was defined as a *p*-value of < 0.5, calculated by one-way analysis of variance (ANOVA) tests at 0 h.

Trans-organ differences were calculated to analyse organ-specific contributions to plasma levels of cf-DNA and cytokines. To calculate a trans-organ difference, the difference in concentration between the efferent and the afferent blood to and from that organ was calculated. The difference between the hepatic vein and the portal vein was designated as a transhepatic difference. The difference between the jugular bulb and the artery was indicated as a transcerebral difference. The difference between the portal vein and the artery was specified as a transsplanchnic difference. Lastly, the difference between the artery and the hepatic vein was classified as a transpulmonary difference.

The group effect in the ANOVA for repeated measures was used in all statistical analyses. One-way ANOVA tests were performed to analyse differences at specific time points. A *p*-value < 0.05 was considered statistically significant. Data with a normal distribution are presented as mean ± standard deviation (±SD) and; data with a non-normal distribution are presented as median and (interquartile range). Statistica™ (Version 13.5, Statsoft, Tulsa, OK) was used in the statistical calculations and for the control of relevant assumptions. A senior statistician was consulted to provide statistical expertise.

## Results

In the animals allocated to group Prot-V after baseline, the levels of cf-DNA in the artery, hepatic vein, portal vein and jugular bulb did not differ between the animals that were ventilated with a V_T_ of 10 mL x kg^− 1^ and those with 6 mL x kg^− 1^ during the preparatory phase of the experiment, Table 1. Because no trend towards a difference materialised in any sample location, defined as a *p* < 0.5, the groups were combined in the analyses according to the statistical plan.

**Table 1:** Cell free DNA (cf-DNA) concentrations (μg x mL^− 1^) at baseline in different sample locations

Sample site	Group		***p***-value
	Prot-7 h	Prot-5 h	
**Artery**	0.54 ± 0.04	0.56 ± 0.09	0.50
**Hepatic vein**	0.54 ± 0.05	0.58 ± 0.19	0.69
**Portal vein**	0.54 ± 0.06	0.52 ± 0.05	0.64
**Jugular bulb**	0.53 ± 0.05	0.54 ± 0.08	0.67

Group Prot-7 h (*n* = 10) was ventilated with a V_T_ of 6 mL x kg^− 1^ throughout the experimentand a PEEP of 5 cmH_2_0 during the preparatory phase but had the PEEP increased to 10 cmH_2_0 after baseline. Group Prot-5 h was ventilated with a V_T_ of 10 mL x kg^− 1^ and a PEEP of 5 cmH_2_0 during the preparatory phase and, after baseline, with a V_T_ of 6 mL x kg^− 1^ and a PEEP of 10 cmH_2_0. Values are given as mean ± SD. The *p*-values are results of one-way ANOVAs

All animals had increased levels of inflammatory cytokines and developed symptoms of a severe systemic inflammatory response to endotoxin infusion with decreasing arterial blood pressure, cardiac index, pulmonary function, and cardiac function. The effects during the experiment in inflammatory cytokines in plasma and group differences have previously been published [27]. The results of the inflammatory cytokines and the physiological responses can be seen in Table 2, without group comparisons.

**Table 2:** Physiological values and levels of inflammatory cytokines during the experiment

Variable	Group	0 h	3 h	5 h
**Temp (°C)**	Prot-V	37.4 ± 1.1	37.9 ± 1.3	37.8 ± 1.3
	Control	37.6 ± 1.3	37.9 ± 1.5	37.9 ± 1.6
**MAP (mmHg)**	Prot-V	91 ± 11	76 ± 23	63 ± 13
	Control	92 ± 9	90 ± 19	71 ± 14
**MPAP (mmHg)**	Prot-V	28 ± 9	40 ± 5	35 ± 7
	Control	25 ± 8	38 ± 4	35 ± 7
**CI (L x min**^**− 1**^**x m**^**− 2**^**)**	Prot-V	2.8 ± 0.5	1.8 ± 0.4	2.0 ± 0.5
	Control	2.6 ± 0.5	2.3 ± 0.4	2.2 ± 0.5
**Lactate (mmol x L**^**−1**^**)**	Prot-V	2.0 ± 0.8	2.6 ± 1.4	2.0 ± 1.0
	Control	2.2 ± 1.0	2.7 ± 1.0	2.5 ± 1.0
**LVSWI (gm x m**^**−2**^ **× beat)**	Prot-V	33 ± 9	16 ± 10	13 ± 5
	Control	32 ± 8	21 ± 7	15 ± 6
**Diuresis (mL x h**^**−1**^**)**	Prot-V	73 (34–190)	25 (15–42)	15 (10–20)
	Control	43 (0–110)	85 (33–110)	11 (4–15)
**PaO2/FiO2 (mmHg)**	Prot-V	478 ± 61	381 ± 107	368 ± 159
	Control	473 ± 70	274 ± 100	219 ± 88
**Trc (10**^**9**^**x L**^**−1**^**)**	Prot-V	388 ± 104	281 ± 107	258 ± 100
	Control	389 ± 93	280 ± 68	255 ± 93
**PEEP (cmH**_**2**_**O)**	Prot-V	5.1 ± 0.2	10.2 ± 0.4	10.1 ± 0.4
	Control	5.0 ± 0	5.0 ± 0	5.0 ± 0
**P peak (cmH**_**2**_**O)**	Prot-V	20 ± 4	25 ± 5	26 ± 5
	Control	20 ± 4	24 ± 3	25 ± 3
**RR (min**^**−1**^**)**	Prot-V	31 ± 15	47 ± 11	49 ± 13
	Control	20 ± 5	22 ± 3	23 ± 5
**VE (L x min**^**−1**^**)**	Prot-V	5.8 ± 1.8	7.0 ± 1.8	7.5 ± 1.9
	Control	5.3 ± 1.4	5.8 ± 1.1	6.1 ± 1.2
**PaCO2 (kPa)**	Prot-V	5.2 ± 0.5	5.6 ± 0.6	5.5 ± 0.5
	Control	5.1 ± 0.4	5.5 ± 0.7	5.6 ± 0.6
**Leukocytes (10**^**9**^**x L**^**−1**^**)**	Prot-V	9 ± 4	10 ± 8	8 ± 5
	Control	10 ± 5	8 ± 5	7 ± 4
**Neutrophils (10**^**9**^**x L**^**−1**^**)**	Prot-V	3 ± 3	6 ± 8	5 ± 5
	Control	4 ± 4	4 ± 4	4 ± 3
**TNF (log**_**10**_**ng x L**^**−1**^**)**	Prot-V	3.3 ± 0.6	3.3 ± 0.2	3.0 ± 0.2
	Control	3.1 ± 0.7	3.6 ± 0.5	3.2 ± 0.4
**IL6 (log**_**10**_**ng x L**^**−1**^**)**	Prot-V	1.8 ± 0.4	2.4 ± 0.3	3.1 ± 0.2
	Control	1.7 ± 0.5	2.6 ± 0.5	3.2 ± 0.3
**IL10 (log**_**10**_**ng x L**^**−1**^**)**	Prot-V	1.9 ± 0.5	1.6 ± 0.2	1.6 ± 0.2
	Control	2.0 ± 0.5	1.8 ± 0.4	1.8 ± 0.5

Group Prot-V (*n* = 20) and group Control (*n* = 10). Values are given as mean ± SD and median (interquartile range). MAP-mean arterial blood pressure, MPAP-mean pulmonary arterial pressure, CI-cardiac index, Lactate-arterial lactate, LVSWI-left ventricular stroke work index, PaO_2_/FiO_2_-ratio of arterial partial pressure of oxygen to fraction of inspired oxygen, P peak-peak intratracheal pressure, RR-respiratory rate, VE-expired minute ventilation, PaCO_2_-arterial partial pressure of carbon dioxide, TNF-α- arterial tumor necrosis factor alpha, IL-6-arterial interleukin 6, IL-10-arterial interleukin 10

### Comparison between cf-DNA levels in different sample locations

**Fig. 2:**
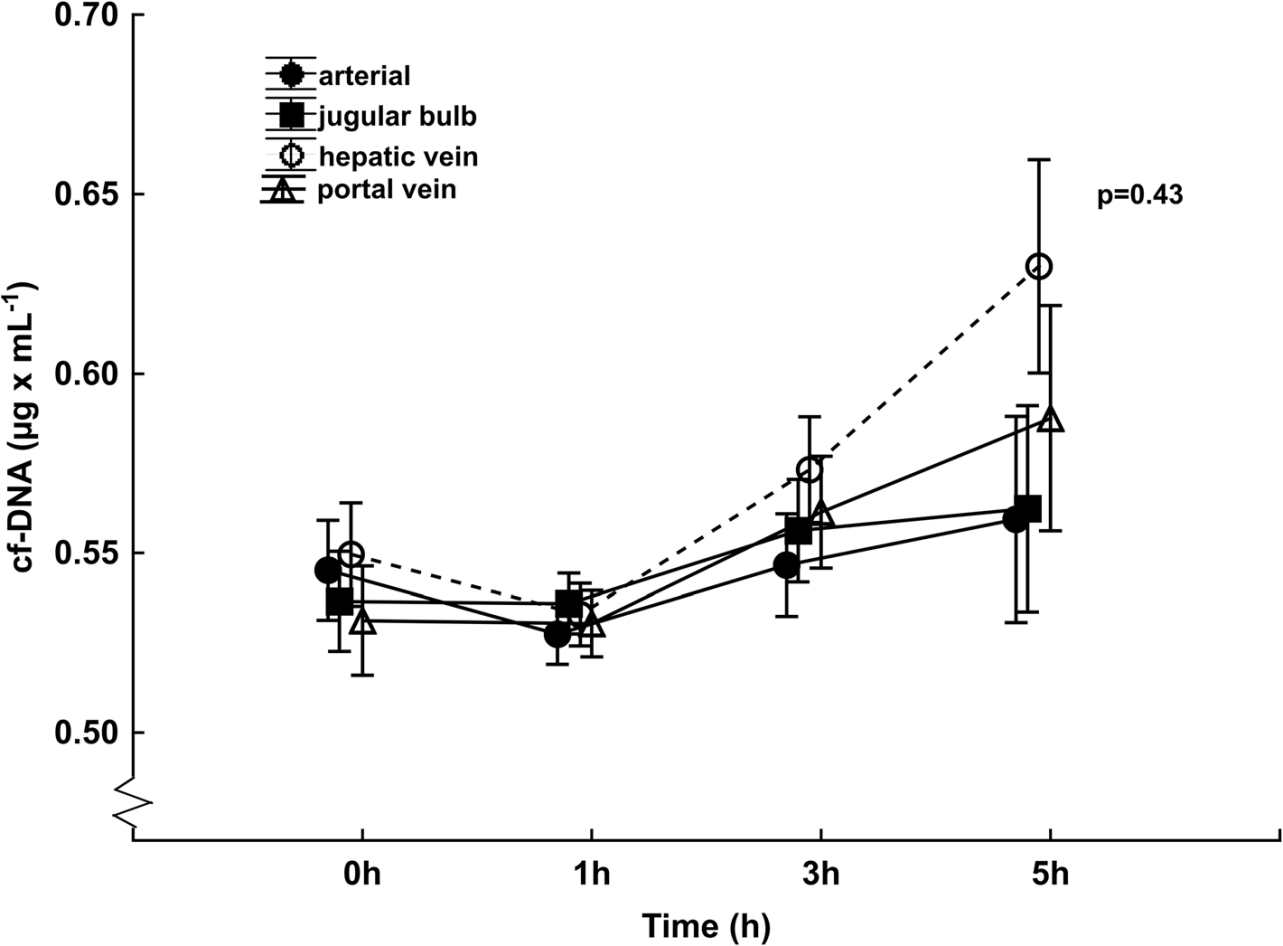
Plasma concentrations of cell free DNA (cf-DNA) in different sampling sites. Values are mean ± SE. The *p*-value is the results of group effect in the analysis of variance (ANOVA) for repeated measures

Plasma concentrations of cf-DNA in different sampling sites are depicted in Fig. 2. In all sampling sites, cf-DNA levels increased as the experiment progressed. The peak levels of cf-DNA were at the experimental endpoint at 5 h. There were no significant differences between the sampling sites, with the hepatic vein having the numerically highest values of all sampling sites.

**Fig. 3:**
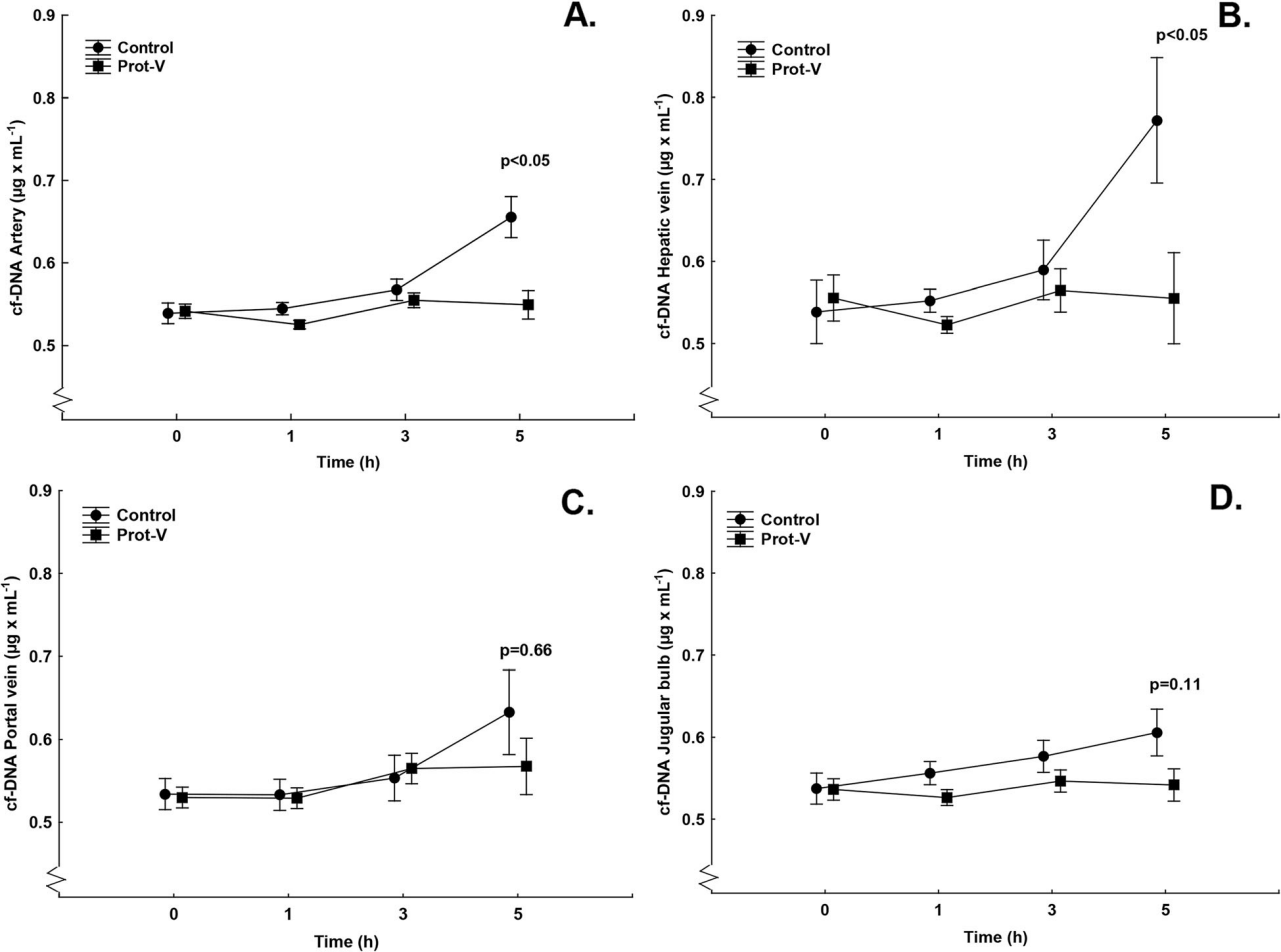
Plasma concentrations of cell free DNA (cf-DNA) by groups. **a**. arterial **b**. hepatic vein **c**. portal vein **d**. jugular bulb. Values are given as mean ± SE. *P*-values are based on the results of group effect in the analysis of variance (ANOVA) for repeated measures

In the artery and the hepatic vein, cf-DNA levels were lower in the group Prot-V at 5 h compared with the group Control (artery, *p* = 0.02; hepatic vein, *p* = 0.03) Fig. 3a-b. No effects of the ventilatory settings on plasma levels of cf-DNA were seen in the portal vein or the jugular bulb, Fig. 3c-d.

### Effect of protective ventilation on trans-organ differences of cf-DNA

**Fig. 4:**
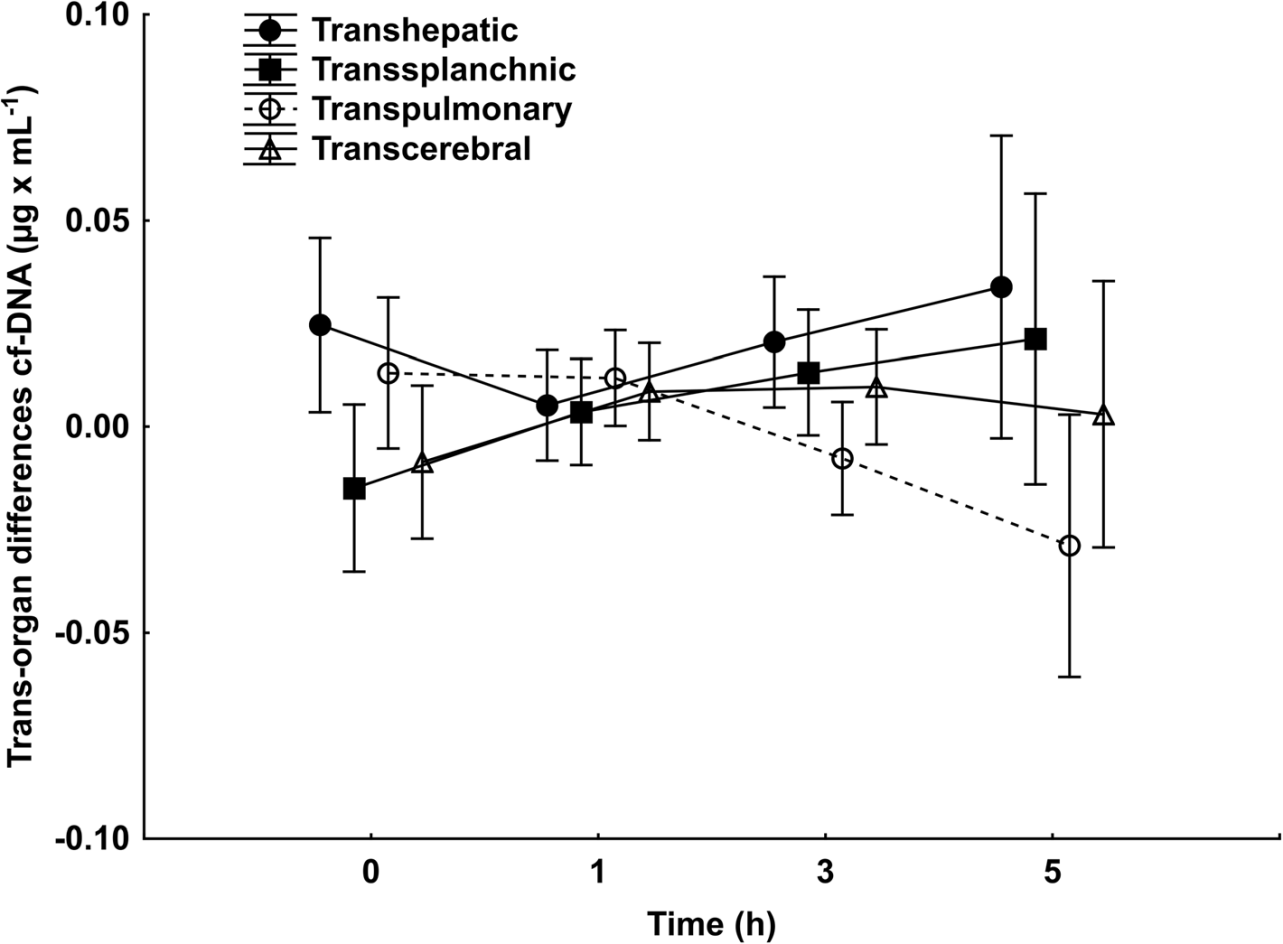
Trans-organ differences in cell free DNA (cf-DNA). Values are given as mean ± SE. *P*-values are based on the results of the group effect in the analysis of variance (ANOVA) for repeated measures. Transpulmonary: the difference in plasma levels between the artery and the hepatic vein, transhepatic: the difference between the hepatic vein and the portal vein, transsplanchnic: the difference between the portal vein and the artery, transcerebral: the difference between the jugular bulb and the artery

**Fig. 5:**
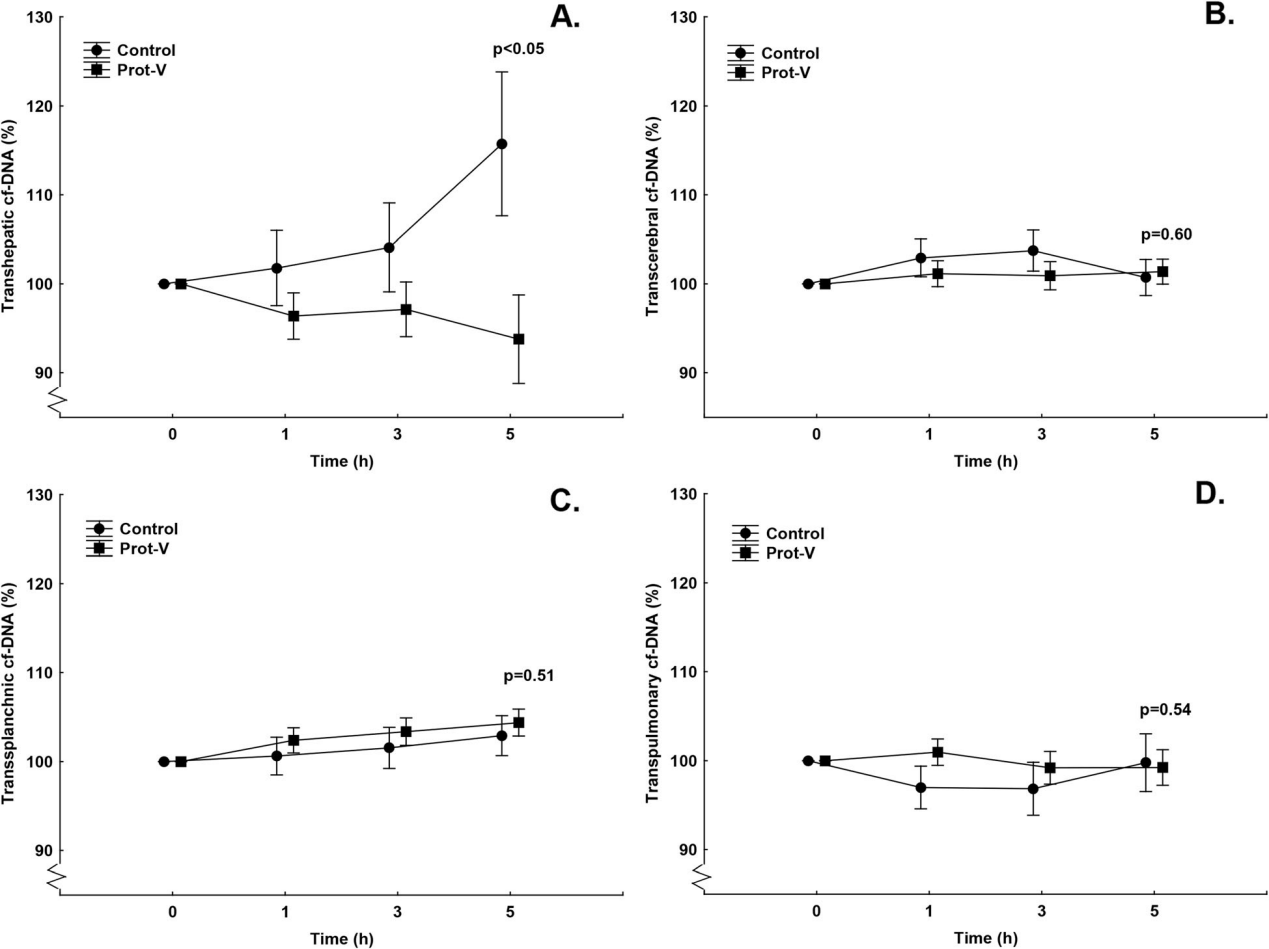
Change from baseline at 0 h (%) in trans-organ differences in cell free DNA (cf-DNA) by groups. **a**. transhepatic **b**. transcerebral **c**. transsplanchnic **d**. transpulmonary. Values are given as mean ± SE. *P*-values are based on the results of the group effect in the analysis of variance (ANOVA) for repeated measures. Transpulmonary: the difference in plasma levels between the artery and the hepatic vein, transhepatic: the difference between the hepatic vein and the portal vein, transsplanchnic: the difference between the portal vein and the artery, transcerebral: the difference between the jugular bulb and the artery

There were no significant differences when the transpulmonary (the difference in plasma levels between the artery and the hepatic vein), transhepatic (the difference between the hepatic vein and the portal vein), transsplanchnic (the difference between the portal vein and the artery) and transcerebral (the difference between the jugular bulb and the artery) differences in cf-DNA were compared, Table 3. Numerically, all trans-organ differences were mainly neutral during the experiment with two exceptions. At the end of the experiment, the transhepatic levels were positive and the transpulmonary levels negative, Fig. 4. The transhepatic cf-DNA differences showed a marked increase in the animals of the grop Control whereas the group Prot-V had an attenuated response, Fig. 5a. No differences between the groups were observed in the transcerebral, transsplanchnic, or the transpulmonary differences, Fig. 5b-d.

**Table 3:** Levels of trans-organ differences in inflammatory cytokines and cell free DNA (cf-DNA)

		Group	0 h	3 h	5 h	p
**TNF-a** (ng x L^−1^)	**Transpulmonary**	Prot-V	− 9222 ± 15,097	− 1293 ± 3383	− 284 ± 645	0.17
		Control	−13,878 ± 10,454	− 3819 ± 1832	− 477 ± 979	
	**Transhepatic**	Prot-V	4919 ± 6573	1275 ± 3394	375 ± 664	0.91
		Control	7531 ± 18,933	3638 ± 4823	− 459 ± 1589	
	**Transcerebral**	Prot-V	2519 ± 6600	369 ± 1749	541 ± 648	0.24
		Control	2154 ± 4868	1560 ± 6843	528 ± 977	
	**Transsplanchnic**	Prot-V	5195 ± 11,969	−34 ± 2080	−27 ± 521	0.22
		Control	4978 ± 13,845	672 ± 968	830 ± 1829	
**IL-6** (ng x L^−1^)	**Transpulmonary**	Prot-V	33 ± 150	990 ± 470	493 ± 424	0.03*
		Control	118 ± 285	1753 ± 1263	2172 ± 632	
	**Transhepatic**	Prot-V	17 ± 105	− 194 ± 258	−49 ± 220	0.19
		Control	−21 ± 65	− 500 ± 282	− 281 ± 242	
	**Transcerebral**	Prot-V	21 ± 183	−62 ± 762	−14 ± 499	0.43
		Control	−16 ± 296	−84 ± 732	− 450 ± 557	
	**Transsplanchnic**	Prot-V	−32 ± 174	− 711 ± 547	− 406 ± 484	0.14
		Control	−126 ± 256	− 1100 ± 1264	− 1646 ± 2600	
**IL-10** (ng x L^−1^)	**Transpulmonary**	Prot-V	−70 ± 81	−30 ± 17	−26 ± 15	0.18
		Control	−138 ± 101	−138 ± 39	−5 ± 100	
	**Transhepatic**	Prot-V	−313 ± 298	−70 ± 63	−5 ± 32	0.31
		Control	− 265 ± 221	−29 ± 109	90 ± 174	
	**Transcerebral**	Prot-V	110 ± 95	32 ± 18	31 ± 20	0.18
		Control	125 ± 83	73 ± 80	34 ± 133	
	**Transsplanchnic**	Prot-V	428 ± 264	91 ± 60	27 ± 30	0.36
		Control	344 ± 174	74 ± 68	61 ± 118	
**cf-DNA** (μg x mL^−1^)	**Transpulmonary**	Prot-V	0.02 ± 0.16	0.01 ± 0.14	0.03 ± 0.17	0.6
		Control	0.01 ± 0.03	−0.04 ± 0.08	−0.17 ± 0.39	
	**Transhepatic**	Prot-V	0.03 ± 0.15	−0.01 ± 0.04	−0.01 ± 0.06	0.03*
		Control	−0.01 ± 0.03	0.04 ± 0.09	0.12 ± 0.04	
	**Transcerebral**	Prot-V	−0.01 ± 0.08	0.01 ± 0.03	0.01 ± 0.03	0.6
		Control	−0.01 ± 0.03	0.03 ± 0.03	−0.01 ± 0.05	
	**Transsplanchnic**	Prot-V	−0.02 ± 0.07	0.01 ± 0.04	0.02 ± 0.07	0.51
		Control	−0.01 ± 0.02	0.01 ± 0.02	0.01 ± 0.05	

Values are given as mean ± SD. *P*-values are results of group effect in the analysis of variance (ANOVA) for repeated measures. * denotes *p* < 0.05. TNF-α-tumor necrosis factor alpha, IL-6-interleukin 6, IL-10-interleukin 10. Transpulmonary: the difference in plasma levels between the artery and the hepatic vein, transhepatic: the difference between the hepatic vein and the portal vein, transsplanchnic: the difference between the portal vein and the artery, transcerebral: the difference between the jugular bulb and the artery

### Effect of protective ventilation on trans-organ differences of cytokines, Table 3

For TNF-α, mean transpulmonary differences were negative during the entire experiment, whereas the mean transhepatic, transsplanchnic, and transcerebral differences were positive. The opposite situation was found for IL-6, where the mean transhepatic, transsplanchnic, and transcerebral differences were negative and the mean transpulmonary differences positive. IL-10 showed negative mean transpulmonary and transhepatic differences and positive mean transsplanchnic and transcerebral differences.

Tidal volume did not affect transpulmonary, transhepatic, transcerebral, or transsplanchnic differences in TNF-α or IL-10. The transpulmonary differences in IL-6 were significantly lower in group Prot-V than in group Control, whereas no differences were seen in transhepatic, transcerebral or transsplanchnic differences.

## Discussion

The main finding of this study is that plasma levels of cf-DNA increase under septic conditions, an effect attenuated by protective ventilation. Additionally, the liver is a significant contributor to the systemic levels of cf-DNA although this effect is also suppressed by protective ventilation with low tidal volumes.

Our study confirms previous results showing that plasma levels of cf-DNA increase during systemic inflammation and sepsis. In addition, our study adds to the evidence that injurious MV affects distant organs [22, 23]. To our knowledge, no study has specifically measured cf-DNA from different sampling sites and how this contributes to the overall levels of cf-DNA; nor has any study accessed how protective ventilation influences these levels under experimental septic conditions. The data suggest that protective ventilation either attenuates the activation of neutrophils, or decreases the amount of cell death; both outcomes would seem beneficial as high levels of cf-DNA carries a poor prognosis in many diseases [1–8].

In one study of patients who died from sepsis in intensive care, immediate post-mortem biopsy revealed extensive hepatic inflammation, centrilobular necrosis and hepatocellular apoptosis in many of these individuals [32]. In an experimental model of shock in mice, Leist et al. demonstrated a causal relationship between TNF-α and liver cell apoptosis [33]. The blood samples in the current experiment was drawn from animals also included in another, already published study [27]. In that study, we showed that the animals in group Prot-V had lower levels of IL-6 and IL-10 in arterial plasma and lower TNF-α and IL-10 in hepatic vein plasma, as compared to the animals in group Control. In the current study we could not deduce a difference in trans-organ cytokine levels in TNF or IL-10, but well lower transpulmonary levels of IL-6 [27].. One plausible explanation of our results is that decreased levels of TNF-α, as a result of protective ventilation, led to a reduction in hepatic cell death, which lowers the level of hepatic and systemic cf-DNA. Our study was not designed to confirm this relationship, however.

Even though the overall levels of cf-DNA increased in the portal circulation, it did not seem to contribute to the systemic output reflected by the transsplanchnic concentrations. This finding is surprising, given that Hotchkiss et al. have shown, both in an experimental model [34] and a clinical study [25], that lymphoid tissue in the intestine and spleen is a major site of apoptosis during sepsis. However, the experimental study had longer observation times and the biopsies included in the clinical study were from deceased patients, which may reflect that apoptosis in these tissues is a later event in the course of sepsis than what we could reproduce with our model. Still, it could suggest that a clearance mechanism of cf-DNA may exist in the gastrointestinal tract. It should also be emphasised that cf-DNA is not solely an idle marker of cell death, rather, cf-DNA exerts its biological effects and has been shown to stimulate the release of pro-inflammatory cytokines [35, 36], activate the coagulation pathway [37, 38], inhibit the fibrinolytic system [39], and cause necrosis of lymphocytes [40].

The clinical implication of this study is that it further adds to the notion that protective ventilation is beneficial for the organism during severe systemic inflammation. In addition, it provides evidence for the theory that the inflammatory output of organs differs during systemic inflammation and sepsis, where the liver seems to have a key role in early inflammation. The above is evident when the trans-organ differences in the inflammatory cytokines in this study are observed. The data suggest that the liver is a net contributor to TNF-α levels, but not to IL-6 and IL-10 levels. The data also indicate that the brain and splanchnic organs are net contributors to the systemic levels of TNF-α and IL-10, whereas the lungs are net contributors to IL-6 levels. The latter effect was significantly attenuated by protective ventilation. To our knowledge this finding has not been described earlier.

The strength of this study is that it is a large animal model, resembling a controlled intensive care setting where inter-individual variability is low, limiting the number of animals needed. The benefit of a porcine model is that the larger animal model resembles human physiology and anatomy, allowing catheter placement that would not be possible in humans, while at the same time requiring a similar approach to fluids and circulatory management as in humans [41, 42]. However, our study also has its limitations. First, the observation time is short. We know that the inflammatory response peaks at 1 to 3 h after starting an endotoxin infusion but organ dysfunction usually peaks towards the end of a 6-h experiment. Hence, the full magnitude of cf-DNA release might not have been observed. Second, it has been suggested that endotoxemia is insufficient to induce the full pathophysiological range of sepsis, but instead, it induces a predictable systemic inflammation, which fulfils the requirement of the study to obtain its goal. Third, to study the transpulmonary differences in cf-DNA and inflammatory cytokines, we were forced to approximate the afferent sample to the hepatic vein. As evident in Fig. 2, the levels of cf-DNA in the hepatic vein were numerically higher than in the other sample sites. The right heart atrium or the pulmonary artery would have been a better measure, but unfortunately no blood samples from those sources were available for analysis. We approximated that the levels of the measured biomarkers in the hepatic vein would most probably be higher than in the venous return from the lower extremities, and thus the approximated transpulmonary differences were likely underestimated. This approximation leads to further evidence of the liver as an important organ in TNF-α and cf-DNA-production during systemic inflammation. Last, no flow measurement devices were placed in organ-specific locations. Hence, we could not relate measured concentrations of cf-DNA to flow and, therefore, we cannot confirm with certainty that the measured levels of cf-DNA in the hepatic vein are in fact signs of increased production.

## Conclusions

In conclusion, in this experimental model of sepsis we showed that lung-protective ventilation suppresses arterial levels of cf-DNA. The liver seems to be a significant contributor to systemic cf-DNA levels, but this effect is attenuated by protective ventilation. The study provides evidence for the theory that the inflammatory output of organs differs during systemic inflammation.

## Data Availability

The dataset supporting the conclusions of this article is available in the Synapse repository, [Synapse ID: syn21162899].

## Abbreviations

ANOVA: Analysis of variance
ARRIVE: Animal Research: Reporting of In Vivo Experiments guidelines
Cf-DNA: Cell-free deoxyribonucleic acid
cm: Centimetres
cmH_2_O: Centimeters of water
CV: Coefficient of variation
F: French
FiO_2_: Fraction of inspired oxygen
h: Hour
I:E: Inspiratory to expiratory ratio
IL-6: Interleukin 6
IL-10: Interleukin 10
i.v.: Intravenous
kg: Kilograms
kPa: Kilopascal
MAP: Mean arterial pressure
mg: Milligrams
mL: Millilitres
MPAP: Mean pulmonary arterial pressure
MODS: Multiorgan dysfunction syndrome
MV: Mechanical ventilation
MQTiPSS: Minimum Quality Threshold in Pre-clinical Sepsis Studies
PEEP: Positive end expiratory pressure
RR: Respiratory rate
PaCO_2_: Partial pressure of carbon dioxide
PaO_2_: Partial pressure of arterial oxygen
SD: Standard deviation
TNF-α: Tumor necrosis factor alpha
VE: Minute ventilation
V_T_: Tidal volume
